# Phosphodiesterase 6 subunits are expressed and altered in idiopathic pulmonary fibrosis

**DOI:** 10.1186/1465-9921-11-146

**Published:** 2010-10-27

**Authors:** Sevdalina Nikolova, Andreas Guenther, Rajkumar Savai, Norbert Weissmann, Hossein A Ghofrani, Melanie Konigshoff, Oliver Eickelberg, Walter Klepetko, Robert Voswinckel, Werner Seeger, Friedrich Grimminger, Ralph T Schermuly, Soni S Pullamsetti

**Affiliations:** 1 University of Giessen Lung Centre (UGLC), Giessen, Germany; 2 Lung Clinic Waldhof Elgershausen, Greifenstein, Germany; 3 Comprehensive Pneumology Center, University Hospital Grosshadern, Ludwig-Maximilians-University, and Helmholtz Zentrum München, Munich, Germany; 4 Department of Cardiothoracic Surgery, University of Vienna, Vienna, Austria; 5 Max-Planck-Institute for Heart and Lung Research, Bad Nauheim, Germany

## Abstract

**Background:**

Idiopathic Pulmonary Fibrosis (IPF) is an unresolved clinical issue. Phosphodiesterases (PDEs) are known therapeutic targets for various proliferative lung diseases. Lung PDE6 expression and function has received little or no attention. The present study aimed to characterize (i) PDE6 subunits expression in human lung, (ii) PDE6 subunits expression and alteration in IPF and (iii) functionality of the specific PDE6D subunit in alveolar epithelial cells (AECs).

**Methodology/Principal Findings:**

PDE6 subunits expression in transplant donor (n = 6) and IPF (n = 6) lungs was demonstrated by real-time quantitative (q)RT-PCR and immunoblotting analysis. PDE6D mRNA and protein levels and PDE6G/H protein levels were significantly down-regulated in the IPF lungs. Immunohistochemical analysis showed alveolar epithelial localization of the PDE6 subunits. This was confirmed by qRT-PCR from human primary alveolar type (AT)II cells, demonstrating the down-regulation pattern of PDE6D in IPF-derived ATII cells. *In vitro*, PDE6D protein depletion was provoked by transforming growth factor (TGF)-β1 in A549 AECs. PDE6D siRNA-mediated knockdown and an ectopic expression of PDE6D modified the proliferation rate of A549 AECs. These effects were mediated by increased intracellular cGMP levels and decreased ERK phosphorylation.

**Conclusions/Significance:**

Collectively, we report previously unrecognized PDE6 expression in human lungs, significant alterations of the PDE6D and PDE6G/H subunits in IPF lungs and characterize the functional role of PDE6D in AEC proliferation.

## Introduction

IPF is a progressive interstitial lung disease of unknown etiology associated with high morbidity and mortality [1], and further characterized by abnormal alveolar epithelial and fibro-proliferative responses, excessive extra-cellular matrix deposition, patchy inflammatory infiltrations and progressive loss of normal lung structure [2]. At present there is no effective therapy for blocking or reversing the progression of the disease [3]. This situation demands a better understanding of the molecular and cellular mechanisms involved in the pathogenesis of IPF.

PDEs comprise a family of related proteins which can be subdivided into 11 families based on their amino acid sequences, sensitivity to different activators and inhibitors and their ability to preferentially hydrolyze either cAMP or cGMP, or both [4]. Of these, PDE6 is a cGMP-specific PDE family and presents multi-component enzyme complexes [5]. The rod PDE6 enzyme is comprised of two catalytic subunits, PDE6α and PDE6β, encoded by the PDE6A and PDE6B genes respectively, two identical inhibitory subunits PDE6γ, encoded by PDE6G [6,7], and one regulatory subunit PDE6δ, encoded by the PDE6D gene [8]. The cone PDE6 enzyme represents two identical catalytic subunits of PDE6α' and two identical inhibitory subunits PDE6γ', encoded by the PDE6C and PDE6H genes, respectively [9]. Primarily localized in the rod and cone photoreceptive cells of the mammalian retina, PDE6 has been widely studied in the context of visual dysfunctions [10,11]. Until now, the expression and characterization of PDE6 in other organs outside of the retina has received little attention. However, recent reports suggest functionality of PDE6 apart from the classical phototransduction cascade [12-14]. PDE6 activity has been coupled to non canonical Wnt5a-Frizzled-2 signaling in non-retinal tissue [12-14]. Recently, a significant increase of Wnt signaling in ATII cells derived from IPF patients and its involvement in epithelial cell injury and hyperplasia has been documented [15].

More interestingly, the specific PDE6D subunit has been reported to regulate the membrane association of Ras and Rap GTPases [16]. The striking similarity between PDE6D and Rho guanine nucleotide dissociation inhibitor (GDI) reasons involvement of PDE6D in cytoskeleton reorganization, membrane trafficking, transcriptional regulation and cell growth control [17]. The study of Cook TA *et al*. demonstrates that PDE6D can modify cGMP hydrolytic activity in preparations of broken rod outer segments [18]. cGMP plays a role in controlling key epithelial cell functions such as ciliary motility, cytokine production and proliferation [19-21].

We therefore hypothesized that i) the PDE6 subunits potentiality can be expressed in the lung, ii) the subunits are differentially regulated in IPF and iii) the specific subunit of PDE6, PDE6D, modulates the proliferation rate of AECs. To this end, we achieved our aim to elucidate previously unrecognized PDE6 expression in normal human lungs, significant alterations of the PDE6D and PDE6G/H subunits in IPF-derived lungs and characterize the functional role of PDE6D in AEC proliferation.

## Materials and methods

### Ethics Statement

The study protocol for tissue donation was approved by the Ethics Committee of the Justus-Liebig-University School of Medicine (AZ 31/93). Informed consent was obtained from each individual patient or the patient's next of kin.

### Human Tissues

Explanted lung tissues from IPF subjects (n = 6) or donor (n = 6) were obtained during lung transplantation at the Department of Cardiothoracic Surgery, University of Vienna, Austria. Diagnosis was established on the basis of a proof of a usual interstitial pneumonitis (UIP) pattern in the explanted lungs from lung transplant recipients [(n = 6; 4 males, 2 females; mean age = 63.33 ± 1.71 yr, mean forced vital capacity (FVC) = 39.00 ± 2.58 (% of standard); mean forced expiratory volume (FEV) = 44.67 ± 6.39 (% of standard); mean carbon monoxide lung diffusion capacity (DL_co_) = 30.5 ± 1.5 (% of predicted)]. Apart from IPF subjects, tissue was also obtained from 6 donor lungs, which could not be utilized due to size limitations between donor and putative recipient (mostly single lobes) or due to incompatibility between donor and recipient.

### Isolation of human ATII cells

Primary human ATII cells were isolated, as previously described [22]. Briefly, the lung was digested and minced. The cell-rich fraction was filtered, layered onto a Percoll density gradient, and centrifuged. The cells were then incubated with anti-CD14 antibody-coated magnetic beads. The remaining cell suspension was incubated in human IgG-coated tissue culture dishes at 37°C in a 5% CO_2_, 95% O_2 _atmosphere. The purity of isolated human ATII cells was examined by Papanicolaou staining. The purity and viability of ATII cell preparations was consistently between 90 and 95%.

### Cell culture

The A549 human AEC line (American Type Culture Collection, Manassas, VA, USA) was maintained in Dulbecco's modified Eagle's (DMEM) F12 medium (Invitrogen, Carlsbad, CA, USA) supplemented with 10% heat-inactivated fetal bovine serum (FBS) (PAA Laboratories GmbH, Pasching, Austria), 100 units/ml penicillin, 0.1 mg/ml streptomycin, and 2 mM L-glutamine at 37°C in a 5% CO_2_, 95% O_2 _atmosphere. For cytokine stimulation A549 cells were cultured in the absence or presence of TGF-β1 (R&D Systems, Minneapolis, USA, final concentration: 2 ng/ml and 5 ng/ml) for 12 h and 24 h. For studies with inhibitors A549 cells were cultured in the absence or presence of ERK inhibitor, U 0126 (Cell Signaling Technology, Beverly, USA, final concentration: 10 μM and 20 μM, solvent: dimethylsulfoxide (DMSO)) or p38α/β inhibitor, SB 203580 (Axon Medchem, Groningen, The Netherlands, final concentration: 10 μM and 20 μM, solvent: DMSO) [23], details are specified in Measurement of Cell proliferation section from Materials and Methods.

### RNA isolation, cDNA synthesis and mRNA quantification by qRT-PCR or semi-quantitative RT-PCR

Total RNA was isolated from frozen human lung tissues and cell pellets using Trizol reagent (Invitrogen, Carlsbad, CA, USA). cDNA synthesis was carried out with an ImProm-II-™ reverse transcription system (Promega Corporation, Madison, WI, USA) by incubating 5 μg of RNA, following the manufacturer's protocol.

qRT-PCR was performed with 2 μl cDNA set up with the Platinum SYBRGreen qPCR SuperMix UDG (Invitrogen, Carlsbad, CA, USA), final volume: 25 μl, using the Mx3000P Real-Time PCR System (Stratagene, La Jolla, CA, USA). Porphobilinogen deaminase (PBGD) and pro-surfactant protein C (SPC), ubiquitously as well as consistently expressed genes were used as reference in total lung homogenates and ATII cells qRT-PCR reactions, respectively. The oligonucleotide primer pairs (human origin): PBGD FP: 5'-TGT CTG GTA ACG GCA ATG CG-3'; RP: 5'-CCCACGCGAATCACTCTCAT-3', pro-SPC FP: 5'-TGA AAC GCC TTC TTA TCG TG-3'; RP: 5'-CTA GTG AGA GCC TCA AGA CTG G-3', PDE6A FP: 5'-TGG CAA AGA GGA CAT CAA AGT-3'; RP: 5'-TAA TCA TCC ATC CAG ACT CAT CC-3', PDE6B FP: 5'-GCA GAA CAA TAG GAA AGA GTG GA-3'; RP: 5'-CAG GAT ACA GCA GGT TGA AGA CT-3', PDE6C FP: 5'-AAG AAT GTT TTG TCC CTG CCT A-3'; RP: 5'-AAG AGT GGC TTT GGT TTG GTT-3', PDE6D FP: 5'-AAT GGT TCT TCG AGT TTG GC-3'; RP: 5'-AAA GTC TCA CTC TGG ATG TGC T-3', PDE6G FP: 5'-TTT AAG CAG CGA CAG ACC AG-3'; RP: 5'-ATA TTG GGC CAG CTC GTG-3', PDE6H FP: 5'-TGA GTG ACA ACA CTA CTC TGC CT-3'; RP: 5'-ATG CAA TTC CAG GTG GCT-3', (final concentration of 200 nM). Relative changes in transcript abundance were expressed as ΔC_T _values (ΔC_T _= DC_T_^reference ^- DC_T_^target^), where higher ΔC_T _values indicate higher transcript abundances [24].

For semi-quantitative RT-PCR 1 μg cDNA was amplified in 50 μl reaction mixture using 0.5 U GoTaq DNA polymerase (Promega, Madison, WI, USA) and 0.5 μM of the following oligonucleotide primer pairs: PDE6A FP: 5'-TGG CAA AGA GGA CAT CAA AGT-3'; RP 5'-TAA TCA TCC ATC CAG ACT CAT CC-3', PDE6B FP: 5'-GCA GAA CAA TAG GAA AGA GTG GA-3'; 5'-CAG GAT ACA GCA GGT TGA AGA CT-3', PDE6C FP: 5'-AAG AAT GTT TTG TCC CTG CCT A-3'; RF: 5'-AAG AGT GGC TTT GGT TTG GTT-3', PDE6D FP: 5'-GGA TGC TGA GAC AGG GAA GAT A-3'; RP: 5'-GCC AGG TAT TTG TGG AGT TAG G-3', PDE6G FP: 5'-GAC AGA CCA GGC AGT TCA AGA G-3'; RP: 5'-TGA GCA GGG TTT AGA GCA CAG T-3', PDE6H FP: 5'-GAC AAC ACT ACT CTG CCT GCT C-3'; RP 5'-GTC ATC TCC AAA TCC TTT CAC AC-3', glyceraldehyde-3-phosphate dehydrogenase (GAPDH) FP: 5'-CAC CGT CAA GGC TGA GAA C-3'; RP: 5'-CAG TAG AGG CAG GGA TGA TGT T-3'. The PCR products were sequence analyzed.

### Immunoblotting

Total protein extracts were isolated from frozen human lung tissues, pig retina and cell pellets homogenized in a lysis buffer containing 150 mM NaCl, 1% Nonidet P-40, 0.1% SDS, 20 mM Tris-HCl pH 7.6, 5 mM EDTA, 1 mM EGTA, 1 mM PMSF and 1× complete mini protease inhibitor cocktail (Roche Diagnostics GmbH, Mannheim, Germany) by centrifugation at 13000 rpm for 20 min at 4^°^C. The protein lysates (25-50 μg) were subjected to SDS-PAGE and immunoblotting for anti-PDE6A, anti-PDE6B, anti-PDE6D, anti-PDE6G/H (FabGennix, Shreveport, LA, USA; Santa Cruz Biotechnology Inc., Heidelberg, Germany, 1:1,000 dilution), anti-His-horseradish peroxidase (HRP) conjugated (Clontech, Heidelberg, Germany, 1: 2,000 dilution), phospho-specific and total anti-ERK (Santa Cruz Biotechnology Inc., Heidelberg, Germany, 1:1,000 dilution), phospho-specific and total anti-p38α/β (Abcam, Cambridge, UK and Cell Signaling Technologies, Danvers, USA, respectively, 1:500 dilution) and anti-GAPDH (Novus Biologicals, Hiddenhausen, Germany, 1:4,000 dilution) antibodies. The signals were visualized using appropriate HRP-conjugated secondary antibodies and developed with an enhanced chemiluminescence (ECL) kit (GE Healthcare UK limited, Buckinghamshire, UK) [25].

#### Blocking with immunizing peptides

Anti-PDE6A and -PDE6B antibodies specificity was validated with PDE6A blocking peptide (M(1)GEVTAEEVEKFLDSN(16)C, Abcam, Cambridge, UK) and PDE6B blocking peptide (H(20)QYFG(K/R)KLSPENVAGAC(36), Abcam, Cambridge, UK), respectively. The signals were developed with an ECL kit as described above. The signal that disappeared when using the blocking peptide (BP) was considered specific to the antibody. GAPDH was used as a control for equal loading.

### Immunohistochemistry

Serial sections of paraffin embedded lung tissue slides (3 μm) were co-stained with anti-PDE6A, anti-PDE6B, anti-PDE6D, anti-PDE6G/H antibodies (Abcam, Cambridge, UK; Proteintech Group Inc., Manchester, UK; Santa Cruz Biotechnology Inc., Heidelberg, Germany, 1:200 dilution) and anti-pro-SPC antibody (Chemicon International Inc., Temecula, CA, USA, 1:1000 dilution). Staining was developed using a rabbit primary amino-ethylcarbazole (AEC) kit (Zymed Laboratories Inc., San Francisco, CA, USA), following the manufacturer's instructions [25].

### Overexpression

For overexpression, the PDE6D gene was PCR amplified from total human lung homogenates by use of platinium high fidelity *Taq *DNA polymerase (Invitrogen, Carlsbad, CA, USA) and oligonucleotide primer pair: FP: 5'-ACC AGA GTG AGA AAG CCG-3' and RP: 5'-CAG TTT CCT CCT CCC TCC AA-3', cloned into the pGEM-T easy vector system (Promega, Madison, WI, USA) and thereafter subcloned into pcDNA3.1/V5-His TOPO eukaryotic expression vector system (Invitrogen, Carlsbad, CA, USA), oligonucleotide primer pair: FP: 5'-CAC CAT GTC AGC CAA GGA C-3; RP: 5'-AAC ATA GAA AAG TCT CAC TCT GGA-3'. Plasmid DNAs for transfection experiments were purified with an endofree plasmid maxi kit (Qiagen, Hilden, Germany).

### siRNA

Endogeneous PDE6D expression in A549 cells was knockdown with PDE6D siRNA target sequence (sense 5'-GGC AGU GUC UCG AGA ACU U-3'; antisense 5'-AAG UUC UCG AGA CAC UGC C-3'; Eurogentec, Seraing, Belgium, 100 nM). Negative control siRNA sequence (Eurogentec, Seraing, Belgium, 100 nM) was used as a specificity control.

### Transient transfection assays

A549 cells were used at 80% confluence. The transient transfection was carried out with Lipofectamine™ 2000 transfection reagent (Invitrogen, Carlsbad, CA, USA) as per the manufacturer's protocol. The transfection efficiency was assessed with anti-PDE6D (FabGennix, Shreveport, LA, USA) and where appropriate with anti-His-HRP conjugated (Clontech, Heidelberg, Germany) antibodies [26].

### Measurement of cell proliferation

A549 cells were transfected under starvation conditions for 6 h, rendered quiescence for 24 h in 0.1% FBS DMEM F12 medium and then subjected to serum stimulation (10% FBS) for 24 h. The effects on cell growth were measured by 3-(4,5-dimethylthiazol-2-yl)-2,5-diphenyltetrazolium bromide (MTT) and [*^3^H*]-Thymidine uptake assay. For studies with inhibitors, A549 cells were rendered quiescence for 24 h in 0.1% FBS DMEM F12 medium and pretreated with U 0126 or SB 203580 for 30 min prior to serum stimulation for 12 h and 24 h. The effects on cell growth were measured by [*^3^H*]-Thymidine uptake assay.

### [^3^H]-Thymidine uptake assay

[^3^H] Thymidine (GE Healthcare UK limited, Buckinghamshire, UK) was used at a concentration 0.1 μCi per well. The [*^3^H*]-Thymidine content of the cell lysates was determined by a scintillation counter (Canberra-Packard, TRI-CARB 2000, Meriden, USA) and the values were expressed as counts per minute (cpm)/number of cells [25]. In addition, cell number was analyzed using the Casy-1 System (Schaerfe, Reutlingen, Germany), based on the Coulter Counter principle.

### PDE activity assay

The A549 cell protein was extracted with RIPA buffer (Santa Cruz, Heidelberg, Germany) and equalized to the same concentration for use. The reactions were performed with 10 μg protein in 100 μl HEPES buffer (40 mM) at pH 7.6 consisting of MgCl_2 _(5 mM), BSA (1 mg/ml) and [^3^H]-cGMP (1 μCi/ml, Amersham Biosciences, Munich, Germany) at 37°C for 15 min. The samples were boiled for 3 min, subsequently cooled for 5 min and incubated with 25 μl Crotalus atrox snake venom (20 mg/ml, Sigma-Aldrich, Munich, Germany) for 15 min at 37°C. After being chilled on ice, the samples were applied to QAE Sephadex A-25 (Amersham Biosciences, Munich, Germany) mini-chromatography columns and eluted with 1 ml ammonium formate (30 mM, pH 7.5). The elutes were collected in 2 ml scintillation solution (Rotiszint^®^eco plus, Roth, Germany) and counted by a beta-counter with CPM (counts per minute) values. Each assay was performed in triplicate and repeated twice independently. Data are expressed as picomoles of cGMP per minute per milligram of protein. (pmol cGMP/minute/mg protein).

### cGMP enzyme immunoassay (EIA)

At the end of culture, cells were washed with PBS twice and lysed in 0.1 M HCl at room temperature for 10 min. After centrifugation the supernatants were equalized to the same protein concentration for use. 50 μl protein samples which were pre-diluted to 0.3 μg/ml and standard solutions were incubated with 50 μl tracer and 50 μl antibody in darkness at 4°C overnight. After washing 5 times, plates were incubated with Ellman's solution for 90-120 min at room temperature with gentle shaking. The plates were read at a wavelength of 405 nm and the concentration was calculated by the ready-made Cayman EIA Double workbook. The standard curve was made as a plot of the %B/B0 value (%Bound/Maximum Bound) vs concentration of a series of known standards using a linear (y) and log (x) axis. Using the 4-parameter logistic equation obtained from the standard curve, the cGMP concentration of samples was determined and is given as nmol/mg protein. Each sample was determined in duplicate and repeated twice.

#### Statistical analysis

All data were expressed as the means ± S.E. Data were compared using a two-tailed Student's *t*-test, or a 1-way ANOVA with the Bonferroni's post hoc test for studies with more than 2 groups. Statistical significance was assumed when *P *< 0.05.

## Results

### mRNA detection of the PDE6 enzyme subunits

**Figure 1:**
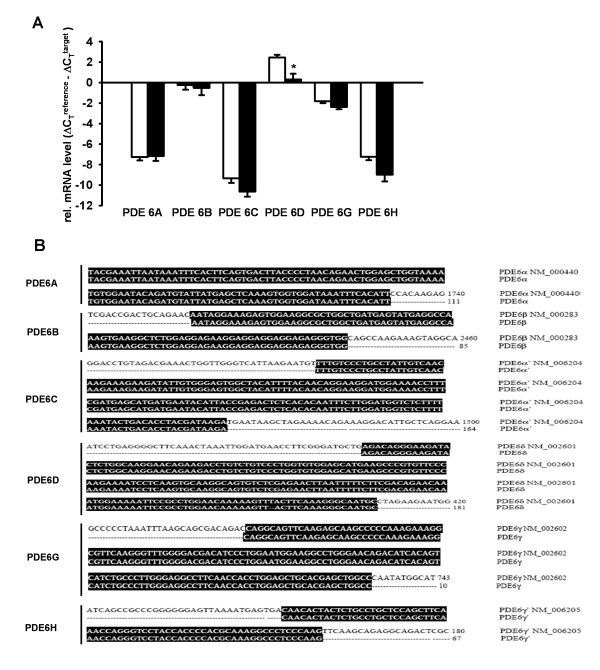
**PDE6 mRNA detection in lung tissues from donors and IPF patients**. *(A) *qRT-PCR analysis was used to assess PDE6 subunits expression in whole lung tissue homogenates from donors (n = 6) and IPF patients (n = 6), white square donor and black square IPF lungs. Each reaction was performed in quadriplicates. Data were present as mean ± S.E, *P < 0.001 versus donor for PDE6D mRNA expression. *(B) *Sequence alignment of the PDE6 subunits.

The mRNA expression of each PDE6 subunit in lung tissue homogenates of donors and IPF patients was analyzed by qRT-PCR technique. As illustrated in Figure 1A, PDE6A, PDE6B, PDE6C, PDE6D, PDE6G and PDE6H mRNAs were expressed in the human lung. PDE6A, PDE6B, PDE6C and PDE6G showed no significant alterations in the IPF lungs as compared to donor lungs. In contrast, PDE6D subunit was significantly down-regulated in the IPF lungs as compared to the donor lungs (relative mRNA expression: 2.44 ± 0.28 and 0.30 ± 0.56, respectively) and PDE6H showed a tendency of down-regulation in the IPF lungs as compared to the donor lungs (relative mRNA expression: -7.22 ± 0.34 and -8.98 ± 0.66, respectively). In addition, the resultant PCR products were validated by direct sequencing, followed by BLAST analysis that confirmed the similar sequence alignment for each subunit (Figure 1B).

### Protein expression of the PDE6 enzyme subunits

**Figure 2:**
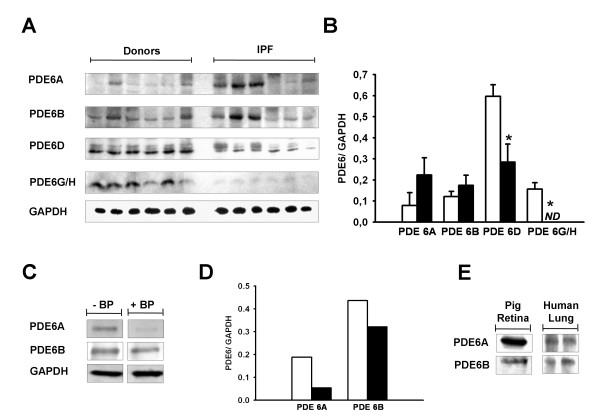
**PDE6 immunoreactivity in lung tissues from donors and IPF patients**. *(A) *Immunoblotting was used to assess PDE6A (~105 kDa), PDE6B (~105 kDa), PDE6D (~17 kDa) and PDE6G/H (~11 kDa) expression in lung tissue homogenates from donors (n = 6) and IPF patients (n = 6). GAPDH (~37 kDa) served as a loading control. *(B) *Corresponding densitometric analysis normalized to GAPDH expression, white square donor and black square IPF lungs. Data were present as mean ± S.E, *P < 0.01 versus donor for PDE6D protein expression and *P < 0.001 versus donor for PDE6G/H protein expression. *(C) *Demonstration of PDE6A and PDE6B antibodies specificity by an antigen (peptide/protein) blocking technique. GAPDH served as a loading control. *(D) *Corresponding densitometric analysis normalized to GAPDH expression, BP (blocking peptide), white square without BP and black square with BP. (E) Immunoblotting showing PDE6A and PDE6B immunoreactivity in pig retina and human lung.

The protein content of the PDE6 subunits in whole lung tissue homogenates of donors and IPF patients was quantified by immunoblotting. As illustrated in Figure 2A, immunoreactivity was detected for PDE6A (~105 kDa), PDE6B (~105 kDa), PDE6D (~17 kDa) and PDE6G/H (~11 kDa) subunits. PDE6A and PDE6B blocking peptide studies were carried out to reconfirm the specificity of PDE6A and PDE6B immunoreactivity (Figure 2C and 2D). Additionally, pig retinal lysate served as a positive control for immunoreactivity and proper protein size (Figure 2E). Notably, the PDE6D and PDE6G/H subunits were significantly down-regulated in the IPF lungs as compared to donor lungs, whereas PDE6A and PDE6B showed no significant alterations between donor and IPF-derived lung tissues (Figure 2B).

### Cellular localization of the PDE6 enzyme subunits

**Figure 3:**
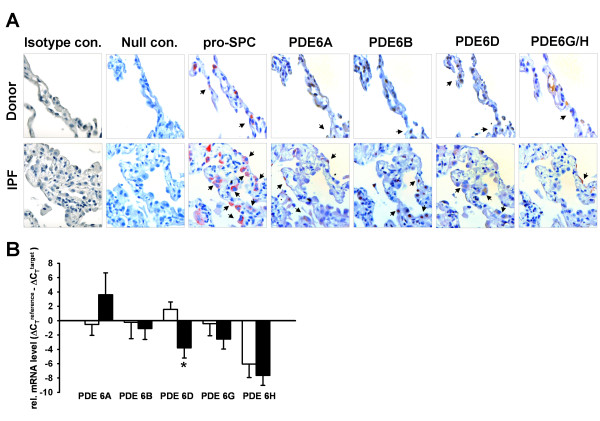
**Cellular and sub-cellular localization of the PDE6 subunits**. *(A) *Immunohistochemical stainings were performed on serial tissue sections of donor (upper row) and IPF (bottom row) lungs. PDE6A, PDE6B, PDE6D and PDE6G/H were co-stained with pro-SPC, a marker specific for ATII cells. PDE6A immunoreactivity was recognized in the cytoplasm and membrane of ATII cells, PDE6B immunoreactivity was recognized in the nuclei, PDE6D immunoreactivity in the cytoplasm and PDE6G/H immunoreactivity in the membrane of ATII cells. The red and dark brown color is indicative of immunoreactivity. Tissue slides were counterstained with hematoxylin (blue color). Isotype control stands for rabbit serum reaction and null control stands for no antibody reaction, magnification 630×. Arrows indicate stained cells. *(B) *PDE6 mRNA expression in human primary donor and IPF-derived ATII cells. Primary human ATII cells were isolated from whole lung tissue of donor and IPF patients as described in Material and Methods. The mRNA levels of PDE6A, PDE6B, PDE6D, PDE6G and PDE6H were analyzed by qRT-PCR. Results are derived from 3 different donor and IPF patients. Each reaction was performed in quadriplicates. Data were present as mean ± S.E, *P < 0.01 versus donor ATII cells.

The cellular localization of the PDE6 subunits was assessed by serial immunohistochemical stainings on tissue sections from donor and IPF lungs. As shown in Figure 3A, PDE6A, PDE6B, PDE6D and PDE6G/H were co-stained with pro-SPC, suggesting the presence of PDE6 subunits in ATII cells. PDE6A immunoreactivity was recognized in the cytoplasm and membrane of ATII cells, PDE6B immunoreactivity was recognized in the nuclei, PDE6D immunoreactivity in the cytoplasm and PDE6G/H immunoreactivity in the membrane of ATII cells.

### PDE6 enzyme subunits expression in human AECs

To confirm the AEC localization pattern, the PDE6 subunits were qRT-PCR amplified from primary human donor and IPF-derived ATII cells. All PDE6 subunits (except for PDE6C, no amplicons were detected by qRT-PCR) were found to be expressed by these cells (Figure 3B). Notably, PDE6D mRNA levels were significantly decreased in IPF-derived ATII cells as compared to donor ATII cells (relative mRNA expression: 1.56 ± 1.05 and -3.80 ± 1.40, respectively). In contrast, PDE6A, PDE6B, PDE6G and PDE6H were not differentially regulated in AECIIs from IPF versus control lungs.

### TGF-β1 down-regulates PDE6D in A549 cells

**Figure 4:**
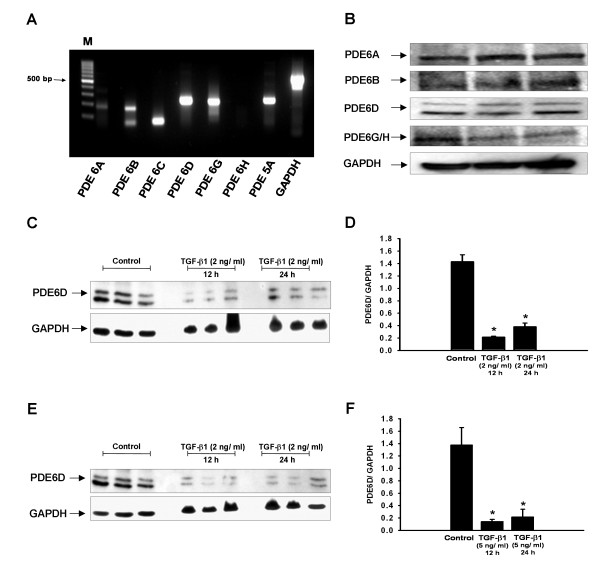
**TGF-β1-induced PDE6D down-regulation in A549 AECs**. *(A) *mRNA expression profile of PDE6 subunits in A549 AECs. *(B) *Protein expression profile of PDE6A (~105 kDa), PDE6B (~105 kDa), PDE6D (~17 kDa) and PDE6G/H (~11 kDa) subunits in A549 AECs. *(C) *TGF-β1 effects on PDE6D expression in A549 cells. A549 cells were rendered quiescence for 24 h in 0.1% FBS DMEM F12 medium, stimulated with TGF-β1 (2 ng/ml) for 12 and 24 h and PDE6D (~17 kDa) expression was measured by immunoblotting. GAPDH (~37 kDa) served as a loading control. *(D) *Corresponding densitometric analysis, normalized to GAPDH expression. Data were present as mean ± S.E, *P < 0.001 versus unstimulated cells. (E) TGF-β1 effects on PDE6D expression in A549 cells. A549 cells were rendered quiescence for 24 h in 0.1% FBS DMEM F12 medium, stimulated with TGF-β1 (5 ng/ml) for 12 and 24 h and PDE6D (~17 kDa) expression was measured by immunoblotting. GAPDH (~37 kDa) served as a loading control. (F) Corresponding densitometric analysis, normalized to GAPDH expression. Data were present as mean ± S.E, *P < 0.01 versus unstimulated cells.

A549 cells were used as an *in vitro *AEC model. Firstly, the cells were characterized for the expression of PDE6 subunits. mRNAs of all PDE6 subunits (except for PDE6C and PDE6H) and the complete set of PDE6 proteins were found to be expressed by these cells (Figure 4A and 4B). Next, to explore whether TGF-β1 promotes PDE6D down-regulation in AECs, A549 cells were treated with two different concentrations of TGF-β1 (2 ng/ml and 5 ng/ml) for 12 and 24 h. Decrease in PDE6D protein expression was clearly evident at concentration as low as 2 ng/ml (Figure 4C and 4D), with no further decrease at higher concentration (5 ng/ml) (Figure 4E and 4F). PDE6D down-regulation occurred within 12 h of TGF-β1 stimulation and was sustained up to 24 h (Figure 4C-F).

### Effects of PDE6D modulations on A549 cells proliferation

**Figure 5:**
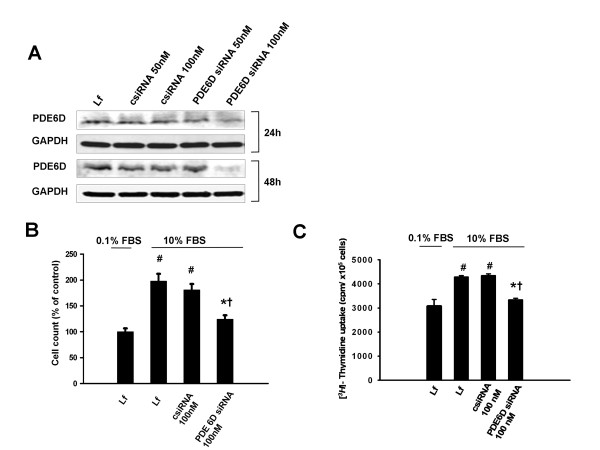
**Knockdown of endogenous PDE6D expression decelerates the proliferation rate of A549 AECs**. *(A) *Demonstration of PDE6D knockdown in A549 cells: upper panel: decreased PDE6D (~17 kDa) immunoreactive protein 0-48 h post transfection with 100 nM PDE6D siRNA. The negative control siRNA (csiRNA, 100 nM) caused no change in PDE6D protein expression. The bottom panel represents GAPDH (~37 kDa) used as a loading control. *(B) *Bar graph presentation of cell counts from PDE6D siRNA transfected cells 24 h post serum stimulation. Data were expressed as % of control. Serum stimulation was significant ^#^*P *< 0.001 versus 0.1% FBS stimulated cells. Cell number from PDE6D knockdown cells was significantly decreased as compared to csiRNA transfected and no siRNA transfected (only lipofectamine (Lf)) cells (*P < 0.001 versus csiRNA 100 nM transfected cells, ^†^*P <*0.01 versus Lf treated cells). *(C) *Bar graph presentation of [*^3^H*]-Thymidine uptake in PDE6D knockdown cells 24 h post serum stimulation. Data were expressed as cpm/×10^5 ^cells. Serum stimulation was significant ^#^*P *< 0.001 versus 0.1% FBS stimulated cells. [*^3^H*]-Thymidine uptake of PDE6D knockdown cells was significantly decreased as compared to csiRNA transfected and Lf treated cells (*P < 0.001 versus csiRNA 100 nM transfected cells, ^†^*P <*0.001 versus Lf treated cells). Lf concentration was kept constant throughout the experimental settings and had no effect on cell viability (P = 0.2699).

**Figure 6:**
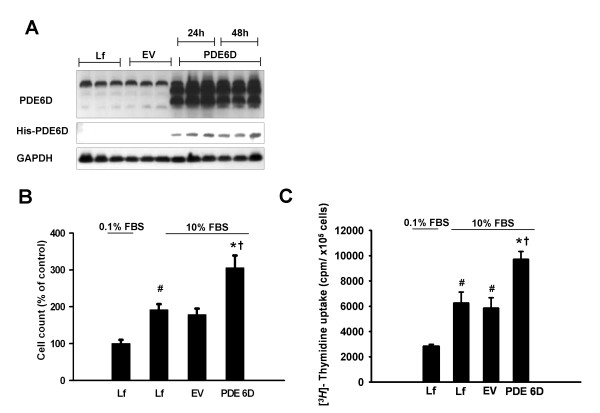
**Overexpression of PDE6D accelerates the proliferation rate of A549 AECs**. *(A) *Demonstration of PDE6D overexpression in A549 cells: upper panel: increased PDE6D (~17 kDa) immunoreactive protein 0-48 h post transfection with pcDNA3.1/His-PDE6D vector (PDE6D). These expressional changes were not observed in pcDNA3.1/His-lacZ empty vector (EV) or no DNA transfected (only lipofectamine (Lf)) cells. Middle panel: The membrane was probed with anti-His-HRP conjugated antibody. A band of ~23 kDa was detected in the PDE6D transfected cells but not in EV transfected or Lf treated cells. The bottom panel represents GAPDH (~37 kDa) used as a loading control. *(B) *Bar graph presentation of cell counts from PDE6D overexpressing cells 24 h post serum stimulation. Data were expressed as % of control. Serum stimulation was significant ^#^*P *< 0.05 versus 0.1% FBS stimulated cells. Cell number from PDE6D overexpressing cells was significantly increased as compared to EV transfected and Lf treated cells (*P < 0.01 versus EV transfected cells, ^†^*P <*0.01 versus Lf treated cells). *(C) *Bar graph presentation of [*^3^H*]-Thymidine uptake in PDE6D overexpressing cells 24 h post serum stimulation. Data were expressed as cpm/×10^5 ^cells. Serum stimulation was significant ^#^*P *< 0.001 versus 0.1% FBS stimulated cells. [*^3^H*]-Thymidine uptake of PDE6D overexpressing cells was significantly increased as compared to EV transfected and Lf treated cells (*P < 0.01 versus EV transfected cells, ^†^*P <*0.01 versus Lf treated cells). Lf concentration was kept constant throughout the experimental settings and had no effect on cell viability (P = 0.3552).

Further, we studied the functional impact of PDE6D modulations on A549 cells proliferation. siRNA silencing of PDE6D resulted in a significant loss of PDE6D protein expression 24 and 48 h post transfection. Transfection with non-targeting siRNA caused no change in PDE6D protein expression (Figure 5A). The loss of PDE6D expression was coupled to a significantly decreased cell number (Figure 5B) and [*^3^H*]-Thymidine uptake (Figure 5C) as compared to control siRNA and no siRNA transfected cells 24 h post serum stimulation. Complementary, transient overexpression of PDE6D in A549 cells resulted in a significantly enhanced PDE6D expression and detection of PDE6D His-tagged protein 24 and 48 h post transfection. Empty vector transfection caused no change in PDE6D protein expression (Figure 6A). The gain of PDE6D expression was coupled to a significantly increased cell number (Figure 6B) and [*^3^H*]-Thymidine uptake (Figure 6C) as compared to empty vector expressing cells and no DNA transfected cells 24 h post serum stimulation.

### PDE6D knockdown regulates cGMP levels and ERK phosphorylation

**Figure 7:**
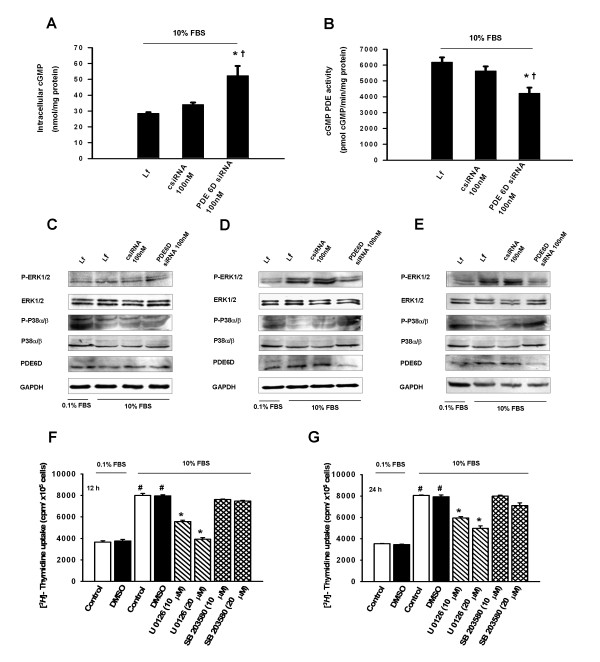
**PDE6D siRNA knockdown inhibits cGMP hydrolyzing PDE activity, increases cGMP levels and inhibits serum stimulated ERK phosphoprylation in A549 AECs**. *(A) *cGMP hydrolyzing PDE activity in PDE6D siRNA transfected cells 24 h post serum stimulation. cGMP PDE activity in PDE6D knockdown cells was significantly decreased as compared to control siRNA (csiRNA) and no siRNA transfected (only lipofectamine (Lf)) cells (*P < 0.05 versus csiRNA 100 nM transfected cells, ^†^*P <*0.01 versus Lf treated cells). *(B) *Intracellular cGMP levels in PDE6D siRNA transfected cells 24 h post serum stimulation. Intracellular cGMP levels were significantly increased as compared to csiRNA transfected and Lf treated cells (*P < 0.01 versus csiRNA 100 nM transfected cells, ^†^*P <*0.01 versus Lf treated cells). *(C, D, E) *Immunoblotting of ERK (~42/44 kDa) and p38α/β (~38/41 kDa) phosphorylation in PDE6D siRNA transfected cells 1 h, 12 h and 24 h post serum stimulation, respectively. GAPDH (~37 kDa) was used as a loading control for PDE6D expression and total ERK (~42/44 kDa) for ERK phosphorylation. *(F, G) *Bar graph presentation of [*^3^H*]-Thymidine uptake in U 0126 (10 μM, 20 μM) and SB 203580 (10 μM, 20 μM) treated cells 12 h and 24 h post serum stimulation, respectively. Serum stimulation was significant ^#^*P *< 0.001 versus 0.1% FBS stimulated cells. [*^3^H*]-Thymidine uptake of U 0126 (10 μM, 20 μM) treated cells was significantly decreased as compared to control (no DMSO) and DMSO treated cells, data were present as mean ± S.E from two independent experiments, *P < 0.001 versus DMSO treated 10% FBS stimulated cells. SB 203580 (10 μM, 20 μM) exerted no effect.

We then opted to explore signaling pathways related to PDE6D-mediated proliferative responses. In particular, we studied the effects of PDE6D down-regulation on (i) cGMP hydrolyzing PDE activity, (ii) intracellular cGMP levels and (iii) serum induced phosphorylation of ERK protein in A549 cells. cGMP hydrolyzing PDE activity was decreased in PDE6D siRNA as compared to non-targeting siRNA and mock transfection 24 h post serum stimulation. In corroboration, intracellular cGMP determined by EIA assay was increased 1.6 fold by PDE6D down-regulation (Figure 7A and 7B). ERK phosphorylation was increased 1 h, 12 h and 24 h post serum stimulation as compared to unstimulated cells (0.1% FBS). siRNA mediated loss of PDE6D protein expression was detectable 12 h and 24 h post serum stimulation and this was related to a decrease in ERK phosphorylation as compared to control siRNA treated cells (Figure 7C-E). However, no apparent changes in the phospho-p38α/β levels were observed by PDE6D down-regulation, suggesting the specificity of PDE6D for ERK signaling (Figure 7C-E).

### ERK inhibition inhibits A549 cells proliferation

Supplementary, employing ERK (U 0126) and p38α/β (SB 203580) pharmacological inhibitors, we showed that ERK1/2 inhibitor (U 0126) significantly inhibits [*^3^H*]-Thymidine uptake 12 h and 24 h post serum stimulation as compared to control (no DMSO) and DMSO treated A549 cells. The effects of U 0126 were dose dependent. Additionally, we used the p38α/β inhibitor (SB 203580) as a control. SB 203580 had no effect on [*^3^H*]-Thymidine uptake by A549 cells (Figure 7F and 7G).

## Discussion

In the present study, we report previously unrecognized PDE6 expression in the human lung. The members of the PDE family, PDE1, PDE2, PDE3, PDE4 and PDE5 are highly expressed in the lung and have been shown to potentially contribute to the pathogenesis of various lung diseases [27,28]. Nevertheless, to our knowledge this is the first report that has described the expression and characterization of PDE6 subunits in both the physiology and pathophysiology of the lung. Among these, PDE6D (mRNA and protein levels) and PDE6G/H subunit (protein levels) were found significantly down-regulated in the IPF lungs as compared to the donor lungs. All PDE6 subunits were detected in ATII cells, with PDE6D significantly down-regulated in IPF-derived ATII cells. PDE6D down-regulation was induced *in vitro *by TGF-β1 in A549 cells, suggesting a link between the observed PDE6D down-regulation in IPF specimens and the pathogenesis of the disease [29]. Furthermore, using A549 cells as an *in vitro *AECs model, we were able to show that PDE6D modulates the proliferation rate of these cells (siRNA and ectopic expression studies). More interestingly, we showed that mechanisms accounting for PDE6D effects on AEC proliferation is related to PDE6D increasing the intracellular cGMP levels and suppressing the phosphorylation of ERK.

This finding is further supported by the reports that have demonstrated solitary PDE6 subunit expression in a variety of non-retinal tissues. The rod catalytic PDE6A and PDE6B subunits were found to be weakly expressed in brain [30,31]. Piriev *et al*. demonstrated that the catalytic core of the rod PDE6 enzyme can be synthesized in human kidney cells with consequent expression of enzymatic activity [32]. The regulatory and the inhibitory PDE6D and PDE6G subunits, respectively, have been reported to be expressed in a variety of heterogeneous tissues, including the lung [8,33]. Identifying the expression of PDE isoforms in organs and cells that had not been reported previously is a subject gaining interest. For example, PDE5, known to express in lung, recently reported to be also expressed in vascular, ganglion and bipolar cell layers of retinal tissue. It was claimed to play a physiological role in the retina and might contribute to PDE5 inhibitor-associated ocular side effects [34].

Although at present the physiological roles of the PDE6 subunits in the lung are unknown and the functionality of the PDE6 enzyme in IPF needs to be explored, the study of Wang *et al*. [14] does provide evidence for the presence of functional PDE6 enzyme in non-retinal tissues. Based on our findings, all the PDE6 subunits appear to be expressed and localize in human lung alveolar epithelium. Among those, PDE6D and PDE6G/H subunit protein levels were found significantly down-regulated in the IPF lungs as compared to the donor lungs, suggesting a plausible contribution of these PDE6 subunits to the pathogenesis of IPF. Thus, we believe that PDE6 alterations may play a crucial role in epithelial apoptosis, proliferation, surfactant synthesis and reactive oxygen species (ROS) generation abnormalities associated with IPF [35].

In fact, based on the data obtained from human donor and IPF lungs, it is not possible at present to determine whether PDE6 functions as a complex or each PDE6 subunit has a solitary function. However, considering the requirement for multiple subunits assembly to produce functional rod and cone PDE6 enzymes and the difficulties in expressing functionally active rod and cone PDE6 enzymes in various systems [36], we herein explored independent functionality of the specific PDE6D subunit in AEC proliferation. In addition, we assessed the contribution of PDE6D to PDE6 as well as to the presence or absence of cGMP. In our studies, gain of function (overexpression) or loss of function (targeting siRNA) of PDE6D affected AEC proliferation, with increased PDE6D resulting in increased AEC proliferation. The anti-proliferative effects encountered in response to PDE6D knockdown were largely due to a decrease in cGMP hydrolyzing PDE activity that may subsequently stimulate the intracellular levels of cGMP. Of note, we were able to measure only total cGMP hydrolyzing activity (Figure 7B), but not PDE6 specific cGMP hydrolyzing activity due to less selectivity of PDE6 inhibitors. Several classes of PDE inhibitors inhibit PDE6 equally as well as the PDE family to which they are targeted [37]. Similarly, further studies are needed to explore the role of PDE6 inhibitory subunits (PDE6G and PDE6H) that were found down regulated at the protein level in IPF lungs. Several lines of evidence reported that the inhibitory PDE6G/H subunits of the PDE6 are expressed in non-retinal tissues [33] and are involved in the stimulation of the p42/p44 mitogen-activated protein kinase (MAPK) pathway by growth factors and G-protein-coupled receptor agonists in human embryonic kidney 293 cells [38].

Impaired AECs proliferation is a significant finding in IPF [39]. Multiple studies have reported rapid proliferation of ATII cells following injury [40,41] or reduced proliferative capacity of ATII cells and inability to differentiate into ATI cells in both experimental lung fibrosis [42] and IPF [39]. Herein, we report modulatory effects of the specific PDE6D subunit on AECs proliferation, as deduced from PDE6D siRNA-mediated knockdown and over-expression studies in A549 cells. This functional property of PDE6D is significant, considering its c-Myc/E2F4 controlled expression (http://www.unleashedinformatics.com). In line with these studies, PDE6D (-/-) mice are consistently smaller in size, indicating a plausible involvement of PDE6D in growth arrest [43]. Thus, it can be imagined that the proliferative phenotype of IPF-derived ATII cells is associated with the observed PDE6D down regulation in IPF lungs.

ERK activation has been shown to be of critical importance for ATII cell proliferation [44]. ERK signaling has also been documented to regulate differentiation of fetal ATII cells [45]. In agreement, our study indicates that ERK is a key mediator of A549 AECs proliferation and that PDE6D mediated proliferative responses are related to ERK signaling. siRNA mediated inhibition of PDE6D decreased the serum induced phosphorylation of ERK in a time response fashion. Thus, we propose PDE6D as a critical regulator of ERK mediated ATII cells proliferation.

In conclusion, these data demonstrate previously unrecognized PDE6 expression in human lung, significant alterations of the PDE6D and PDE6G/H subunits in IPF-derived lungs and characterize the functional role of PDE6D in AEC proliferation. For a further consolidation of the proposed pathomechanistic link between PDE6D content and type II cell proliferation on an *in vivo *level, transgenic mice with epithelial cell-specific PDE6D knockout would have to be generated. Hence, we can, right now, only postulate that decreased PDE6D expression in IPF might be involved in attenuation of type II cell hyperplasia. Further, it is tempting to speculate that therapeutic prevention of PDE6D down-regulation and/or PDE6D overexpression in animal models of pulmonary fibrosis may be beneficial to boost up alveolar re-epithelization and may represent a therapeutic option in IPF.

## Competing interests

The authors declare that they have no competing interests.

## Authors' contributions

Conceived and designed the experiments: SN, NW, HAG, RTS, SSP. Performed the experiments: SN, RS, SSP. Analyzed the experiments: AG, WS, FG. Contributed reagents/Materials: MK, OE, WK, RV. Wrote the paper: SN, RTS, SSP. All authors read and approved the manuscript.

## References

[B1] American Thoracic Society Idiopathic pulmonary fibrosis: diagnosis and treatment. International consensus statement. American Thoracic Society (ATS), and the European Respiratory Society (ERS) Am J Respir Crit Care Med 2000 161 2 Pt 1 646 664 10673212 10.1164/ajrccm.161.2.ats3-00

[B2] Selman M King TE Pardo A Idiopathic pulmonary fibrosis: prevailing and evolving hypotheses about its pathogenesis and implications for therapy Ann Intern Med 2001 134 2 136 151 11177318 10.7326/0003-4819-134-2-200101160-00015

[B3] Thannickal VJ Flaherty KR Hyzy RC Lynch JP Emerging drugs for idiopathic pulmonary fibrosis Expert Opin Emerg Drugs 2005 10 4 707 727 10.1517/14728214.10.4.707 16262559

[B4] Beavo JA Cyclic nucleotide phosphodiesterases: functional implications of multiple isoforms Physiol Rev 1995 75 4 725 748 7480160 10.1152/physrev.1995.75.4.725

[B5] Cote RH Characteristics of photoreceptor PDE (PDE6): similarities and differences to PDE5 Int J Impot Res 2004 16 Suppl 1 S28 33 10.1038/sj.ijir.3901212 15224133

[B6] Baehr W Devlin MJ Applebury ML Isolation and characterization of cGMP phosphodiesterase from bovine rod outer segments J Biol Chem 1979 254 22 11669 11677 227876

[B7] Deterre P Bigay J Forquet F Robert M Chabre M cGMP phosphodiesterase of retinal rods is regulated by two inhibitory subunits Proc Natl Acad Sci USA 1988 85 8 2424 2428 10.1073/pnas.85.8.2424 2833739 PMC280009

[B8] Lorenz B Migliaccio C Lichtner P Meyer C Strom TM D'Urso M Becker J Ciccodicola A Meitinger T Cloning and gene structure of the rod cGMP phosphodiesterase delta subunit gene (PDED) in man and mouse Eur J Hum Genet 1998 6 3 283 290 10.1038/sj.ejhg.5200215 9781033

[B9] Gillespie PG Beavo JA Characterization of a bovine cone photoreceptor phosphodiesterase purified by cyclic GMP-sepharose chromatography J Biol Chem 1988 263 17 8133 8141 2836413

[B10] Muradov KG Granovsky AE Artemyev NO Mutation in rod PDE6 linked to congenital stationary night blindness impairs the enzyme inhibition by its gamma-subunit Biochemistry 2003 42 11 3305 3310 10.1021/bi027095x 12641462

[B11] Stockman A Sharpe LT Tufail A Kell PD Ripamonti C Jeffery G The effect of sildenafil citrate (Viagra) on visual sensitivity J Vis 2007 7 8 4 10.1167/7.8.4 17685811

[B12] Ahumada A Slusarski DC Liu X Moon RT Malbon CC Wang HY Signaling of rat Frizzled-2 through phosphodiesterase and cyclic GMP Science 2002 298 5600 2006 2010 10.1126/science.1073776 12471263

[B13] Ma L Wang HY Mitogen-activated protein kinase p38 regulates the Wnt/cyclic GMP/Ca2+ non-canonical pathway J Biol Chem 2007 282 39 28980 28990 10.1074/jbc.M702840200 17684012

[B14] Wang H Lee Y Malbon CC PDE6 is an effector for the Wnt/Ca2+/cGMP-signalling pathway in development Biochem Soc Trans 2004 32 Pt 5 792 796 15494017 10.1042/BST0320792

[B15] Konigshoff M Balsara N Pfaff EM Kramer M Chrobak I Seeger W Eickelberg O Functional Wnt signaling is increased in idiopathic pulmonary fibrosis PLoS ONE 2008 3 5 e2142 10.1371/journal.pone.0002142 18478089 PMC2374879

[B16] Nancy V Callebaut I El Marjou A de Gunzburg J The delta subunit of retinal rod cGMP phosphodiesterase regulates the membrane association of Ras and Rap GTPases J Biol Chem 2002 277 17 15076 15084 11786539 10.1074/jbc.M109983200

[B17] Van Aelst L D'Souza-Schorey C Rho GTPases and signaling networks Genes Dev 1997 11 18 2295 2322 10.1101/gad.11.18.2295 9308960

[B18] Cook TA Ghomashchi F Gelb MH Florio SK Beavo JA The delta subunit of type 6 phosphodiesterase reduces light-induced cGMP hydrolysis in rod outer segments J Biol Chem 2001 276 7 5248 5255 10.1074/jbc.M004690200 11053432

[B19] Friedman DL Role of cyclic nucleotides in cell growth and differentiation Physiol Rev 1976 56 4 652 708 185633 10.1152/physrev.1976.56.4.652

[B20] Geary CA Davis CW Paradiso AM Boucher RC Role of CNP in human airways: cGMP-mediated stimulation of ciliary beat frequency Am J Physiol 1995 268 6 Pt 1 L1021 1028 7611424 10.1152/ajplung.1995.268.6.L1021

[B21] Stadnyk AW Cytokine production by epithelial cells FASEB J 1994 8 13 1041 1047 7926369 10.1096/fasebj.8.13.7926369

[B22] Fang X Song Y Hirsch J Galietta LJ Pedemonte N Zemans RL Dolganov G Verkman AS Matthay MA Contribution of CFTR to apical-basolateral fluid transport in cultured human alveolar epithelial type II cells Am J Physiol Lung Cell Mol Physiol 2006 290 2 L242 249 10.1152/ajplung.00178.2005 16143588

[B23] Ogura H Tsukumo Y Sugimoto H Igarashi M Nagai K Kataoka T ERK and p38 MAP kinase are involved in downregulation of cell surface TNF receptor 1 induced by acetoxycycloheximide Int Immunopharmacol 2008 8 6 922 926 10.1016/j.intimp.2008.02.010 18442799

[B24] Pullamsetti S Kiss L Ghofrani HA Voswinckel R Haredza P Klepetko W Aigner C Fink L Muyal JP Weissmann N Increased levels and reduced catabolism of asymmetric and symmetric dimethylarginines in pulmonary hypertension FASEB J 2005 19 9 1175 1177 15827267 10.1096/fj.04-3223fje

[B25] Schermuly RT Dony E Ghofrani HA Pullamsetti S Savai R Roth M Sydykov A Lai YJ Weissmann N Seeger W Reversal of experimental pulmonary hypertension by PDGF inhibition J Clin Invest 2005 115 10 2811 2821 10.1172/JCI24838 16200212 PMC1236676

[B26] Hanze J Eul BG Savai R Krick S Goyal P Grimminger F Seeger W Rose F RNA interference for HIF-1alpha inhibits its downstream signalling and affects cellular proliferation Biochem Biophys Res Commun 2003 312 3 571 577 10.1016/j.bbrc.2003.10.153 14680803

[B27] Schermuly RT Pullamsetti SS Kwapiszewska G Dumitrascu R Tian X Weissmann N Ghofrani HA Kaulen C Dunkern T Schudt C Phosphodiesterase 1 upregulation in pulmonary arterial hypertension: target for reverse-remodeling therapy Circulation 2007 115 17 2331 2339 10.1161/CIRCULATIONAHA.106.676809 17438150

[B28] Galie N Ghofrani HA Torbicki A Barst RJ Rubin LJ Badesch D Fleming T Parpia T Burgess G Branzi A Sildenafil citrate therapy for pulmonary arterial hypertension N Engl J Med 2005 353 20 2148 2157 10.1056/NEJMoa050010 16291984

[B29] Bergeron A Soler P Kambouchner M Loiseau P Milleron B Valeyre D Hance AJ Tazi A Cytokine profiles in idiopathic pulmonary fibrosis suggest an important role for TGF-beta and IL-10 Eur Respir J 2003 22 1 69 76 10.1183/09031936.03.00014703 12882453

[B30] Kuenzi F Rosahl TW Morton RA Fitzjohn SM Collingridge GL Seabrook GR Hippocampal synaptic plasticity in mice carrying the rd mutation in the gene encoding cGMP phosphodiesterase type 6 (PDE6) Brain Res 2003 967 1-2 144 151 10.1016/S0006-8993(02)04241-5 12650975

[B31] Taylor RE Shows KH Zhao Y Pittler SJ A PDE6A promoter fragment directs transcription predominantly in the photoreceptor Biochem Biophys Res Commun 2001 282 2 543 547 10.1006/bbrc.2001.4605 11401494

[B32] Piriev NI Yamashita C Samuel G Farber DB Rod photoreceptor cGMP-phosphodiesterase: analysis of alpha and beta subunits expressed in human kidney cells Proc Natl Acad Sci USA 1993 90 20 9340 9344 10.1073/pnas.90.20.9340 8415703 PMC47563

[B33] Tate RJ Arshavsky VY Pyne NJ The identification of the inhibitory gamma-subunits of the type 6 retinal cyclic guanosine monophosphate phosphodiesterase in non-retinal tissues: differential processing of mRNA transcripts Genomics 2002 79 4 582 586 10.1006/geno.2002.6740 11944991

[B34] Foresta C Caretta N Zuccarello D Poletti A Biagioli A Caretti L Galan A Expression of the PDE5 enzyme on human retinal tissue: new aspects of PDE5 inhibitors ocular side effects Eye 2008 22 1 144 149 10.1038/sj.eye.6702908 17585311

[B35] Horowitz JC Thannickal VJ Idiopathic pulmonary fibrosis: new concepts in pathogenesis and implications for drug therapy Treat Respir Med 2006 5 5 325 342 10.2165/00151829-200605050-00004 16928146 PMC2231521

[B36] Ionita MA Pittler SJ Focus on molecules: rod cGMP phosphodiesterase type 6 Exp Eye Res 2007 84 1 1 2 10.1016/j.exer.2005.12.012 16563379 PMC10546788

[B37] Zhang X Feng Q Cote RH Efficacy and selectivity of phosphodiesterase-targeted drugs in inhibiting photoreceptor phosphodiesterase (PDE6) in retinal photoreceptors Invest Ophthalmol Vis Sci 2005 46 9 3060 3066 10.1167/iovs.05-0257 16123402 PMC1343468

[B38] Wan KF Sambi BS Frame M Tate R Pyne NJ The inhibitory gamma subunit of the type 6 retinal cyclic guanosine monophosphate phosphodiesterase is a novel intermediate regulating p42/p44 mitogen-activated protein kinase signaling in human embryonic kidney 293 cells J Biol Chem 2001 276 41 37802 37808 11502744 10.1074/jbc.M105087200

[B39] Kasper M Haroske G Alterations in the alveolar epithelium after injury leading to pulmonary fibrosis Histol Histopathol 1996 11 2 463 483 8861769

[B40] Stephens RJ Sloan MF Evans MJ Freeman G Early response of lung to low levels of ozone Am J Pathol 1974 74 1 31 58 4809315 PMC1910726

[B41] Evans MJ Cabral LJ Stephens RJ Freeman G Renewal of alveolar epithelium in the rat following exposure to NO2 Am J Pathol 1973 70 2 175 198 4566990 PMC1903972

[B42] Adamson IY Young L Bowden DH Relationship of alveolar epithelial injury and repair to the induction of pulmonary fibrosis Am J Pathol 1988 130 2 377 383 3341452 PMC1880524

[B43] Zhang H Li S Doan T Rieke F Detwiler PB Frederick JM Baehr W Deletion of PrBP/delta impedes transport of GRK1 and PDE6 catalytic subunits to photoreceptor outer segments Proc Natl Acad Sci USA 2007 104 21 8857 8862 10.1073/pnas.0701681104 17496142 PMC1885592

[B44] Thrane EV Schwarze PE Thoresen GH Lag M Refsnes M Persistent versus transient map kinase (ERK) activation in the proliferation of lung epithelial type 2 cells Exp Lung Res 2001 27 4 387 400 10.1080/019021401750193638 11400863

[B45] Sanchez-Esteban J Wang Y Gruppuso PA Rubin LP Mechanical stretch induces fetal type II cell differentiation via an epidermal growth factor receptor-extracellular-regulated protein kinase signaling pathway Am J Respir Cell Mol Biol 2004 30 1 76 83 10.1165/rcmb.2003-0121OC 12829451

