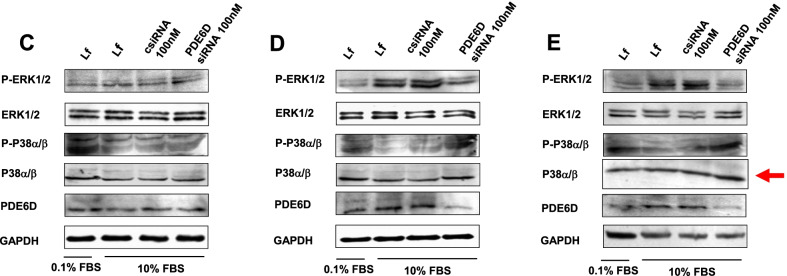# Correction to: Phosphodiesterase 6 subunits are expressed and altered in idiopathic pulmonary fibrosis

**DOI:** 10.1186/s12931-021-01907-5

**Published:** 2022-01-06

**Authors:** Sevdalina Nikolova, Andreas Guenther, Rajkumar Savai, Norbert Weissmann, Hossein A. Ghofrani, Melanie Konigshoff, Oliver Eickelberg, Walter Klepetko, Robert Voswinckel, Werner Seeger, Friedrich Grimminger, Ralph T. Schermuly, Soni S. Pullamsetti

**Affiliations:** 1 University of Giessen Lung Centre (UGLC), Giessen, Germany; 2 Lung Clinic Waldhof Elgershausen, Greifenstein, Germany; 3 Comprehensive Pneumology Center, University Hospital Grosshadern, Ludwig-Maximilians-University, and Helmholtz Zentrum München, Munich, Germany; 4 Department of Cardiothoracic Surgery, University of Vienna, Vienna, Austria; 5 Max-Planck-Institute for Heart and Lung Research, Bad Nauheim, Germany

## Correction to: Respir Res (2010) 11:146 http://respiratory-research.com/content/11/1/146

The original version of the article [1] unfortunately contained a mistake in Fig. 7.

In this a western blot image (p38a/b, panel E in Fig. 7) from the 24 h PDE6D siRNA transfected group has been mistakenly duplicated from the 12 h PDE6D siRNA transfected group shown in Fig. 7D.

We are deeply sorry for this mistake and would like to replace it with a blot from 24 h PDE6D siRNA transfected group. This change will not affect the results, discussion, and the general message of the manuscript.

It has been corrected in this correction.

The correct version of Fig. 7 is in this erratum.